# Association between **ε**2/3/4, Promoter Polymorphism (−491A/T, −427T/C, and −219T/G) at the Apolipoprotein E Gene, and Mental Retardation in Children from an Iodine Deficiency Area, China

**DOI:** 10.1155/2014/236702

**Published:** 2014-03-25

**Authors:** Jun Li, Fuchang Zhang, Yunliang Wang, Yan Wang, Wei Qin, Qinghe Xing, Xueqing Qian, Tingwei Guo, Xiaocai Gao, Lin He, Jianjun Gao

**Affiliations:** ^1^Bio-X Institute, Key Laboratory for the Genetics of Developmental and Neuropsychiatric Disorders (Ministry of Education), Shanghai Jiao Tong University, 1954 Huashan Road, Shanghai 200030, China; ^2^Institute for Neuropsychiatric Science and Metabonomics, Changning Mental Health Center, Shanghai 200335, China; ^3^Key Laboratory of Resource Biology and Biotechnology in Western China (Ministry of Education), College of Life Science, Institute of Population and Health, Northwest University, Xi'an 710069, China; ^4^Department of Neurology, the 148th Hospital, Zibo, Shandong 255300, China; ^5^Shanghai Institute of Planned Parenthood Research, 200013, Shanghai, China; ^6^Institute for Nutritional Sciences, Shanghai Institutes for Biological Sciences, Chinese Academy of Sciences, Graduate School of Chinese Academy Sciences, Shanghai 200031, China

## Abstract

*Background.* Several common single-nucleotide polymorphisms (SNPs) at apolipoprotein E (ApoE) have been linked with late onset sporadic Alzheimer's disease and declining normative cognitive ability in elder people, but we are unclear about their relationship with cognition in children. *Results.* We studied −491A/T, −427T/C, and −219G/T promoter polymorphisms and *ε*2/*ε*3/*ε*4 at ApoE among children with mental retardation (MR, *n* = 130), borderline MR (*n* = 124), and controls (*n* = 334) from an iodine deficiency area in China. The allelic and genotypic distribution of individual locus did not significantly differ among three groups with Mantel-Haenszel *χ* ^2^ test (*P* > 0.05). However, frequencies of haplotype of −491A/−427T/−219T/*ε*4 were distributed as MR > borderline MR > controls (*P* uncorrected = 0.004), indicating that the presence of this haplotype may increase the risk of disease. *Conclusions.* In this large population-based study in children, we did not find any significant association between single locus of the four common ApoE polymorphisms (−491A/T, −427T/C, −219T/G, and *ε*2/3/4) and MR or borderline MR. However, we found that the presence of ATT*ε*4 haplotype was associated with an increased risk of MR and borderline MR. Our present work may help enlarge our knowledge of the cognitive role of ApoE across the lifespan and the mechanisms of human cognition.

## 1. Introduction

Mental retardation (MR) is a condition of neuropsychiatric dysfunction featured by an impairment of intellectual abilities and a deficit of adaption to the environment and the social milieu [1]. Among the complex potential causes of MR, iodine deficiency constitutes the world's greatest single cause of preventable MR [2]. Children born in iodine deficient areas are at great risk for loss of intelligence quotient (IQ) caused by the combined effects of maternal, fetal, and neonatal hypothyroxinemia [3].

Familial aggregation of MR is common in the Qin-Ba Mountain region (Qinba) in midwestern China. Our previous investigation demonstrated that nonspecific mental retardation (NSMR) in Qinba has a heritability with 58.2% (Sd = 19.3%) and may be determined by a lot of minor effective genes [4]. Numeric associations between general intelligence and specific single-nucleotide polymorphisms (SNPs) in several candidate genes involved in brain function have been reported [5, 6]. Apolipoprotein E (ApoE) plays an important role in cholesterol transport and plasma lipoprotein metabolism [7]. Several overlapping functions have been attributed to ApoE potentially, such as lipid transport, neuronal repair, dendritic growth, maintenance of synaptic plasticity, and anti-inflammatory activities [8]. Its potential role in brain functioning is wide ranging. The common variations of ApoE-*ε*2/3/4, defined by two common SNPs (rs7412 for ApoE *ε*2 and rs429358 for ApoE *ε*4) [9], are the best established susceptibility gene for late-onset Alzheimer's disease (AD) [10, 11]. They were also linked with declining cognitive ability in older people without Alzheimer's disease [12]. The hypothesis that ApoE *ε*4 is also associated with cognitive decline in old age has been investigated in a few studies with inconsistent results. Despite the existing inconsistency, accumulating evidence or stronger effect on intelligence was found in the older people; by contrast, fewer pieces of evidence were found in younger people. Recently, a few studies have examined the association between cognition and the common ApoE polymorphisms in children or younger adults, but the results were inconsistent [13, 14].

In addition to the ApoE-*ε*2/*ε*3/*ε*4 polymorphisms in coding sequence, other common polymorphisms in promoter including −491A/T (numbered relatively to the transcription start site, rs449647), −427T/C (rs769446), and −219G/T (rs405509) were linked with the quantitative expression of ApoE [15, 16] and associated with AD [17–22]. However, to the best of our knowledge, only one study has looked into whether these polymorphisms are related to intelligence in children individually [13]. No study has investigated the haplotype constituting the polymorphisms in promoter and ApoE-*ε*2/3/4. Their relationship to intelligence at an early age is a thought of particular interest, because it would potentially aid in understanding the role of ApoE for cognitive functioning across the lifespan [14, 23] and the nature of human cognitive mechanisms.

## 2. Materials and Methods

### 2.1. Subjects

Between 1995 and 1998, we carried out an investigation on MR in two areas with high risk in Qinba Mountains, Zhashui county and Ankang county. Among the two counties, some typical villages with high risk of MR were selected as the target areas in our investigation. All children aged 0–14 years from these areas (*n* = 2974 in Zhashui and *n* = 2178 in Ankang) were recruited and then screened by different instruments/scales. Details about the study design can be found elsewhere [24]. Briefly, based on the* Chinese Classification of Mental Disorders 2nd Revision* and the classification of mental and behavioral disorders from the World Health Organization (WHO), the clinical psychiatric pediatricians diagnosed, identified, and classified the MR or borderline MR in the Qinba region of China. We also drew family pedigrees to investigate if there existed possible familial mental retardation. All subjects were Han Chinese in origin. We also collected peripheral blood samples from children aged 6–14 years because they were more collaborated. Blood specimens were saved under −70°C until the analysis. The protocol was reviewed and approved by the Ethical Committee of the National Human Genome Center. The guardians of all participated children provided written informed consent.

### 2.2. Assessment of Mental Retardation

For children aged 6–14 years, we first screened their intelligence using Chinese Standardization of Raven's Standard Progressive Matrices [25]. If children's IQ <85, we assessed their adaptive behavior using Chinese Revised Scale of Social Adaption Ability of Infant-Junior Middle School Student [26]. For children with scores of adaption ability ≤9, we reassessed their intelligence with Chinese-Wechsler Intelligence Scale for Children [27]. MR or borderline MR was finally diagnosed by professional assessment of intelligence and adaptive behavior according to the International Classification of Diseases-10 (1990, WHO).

### 2.3. Genotyping

A total of 588 blood samples were drawn from children aged 6–14 years with MR (*n* = 130), borderline MR (*n* = 124), and non-MR (controls, *n* = 334).

Leukocyte DNA was extracted using a standard phenol/chloroform method. ApoE promoter polymorphisms −491A/T, −427T/C, and −219G/T were genotyped via a nested PCR amplification. Firstly, the parent 1426 bp fragment was amplified using the primers 5′-CAAGGTCACACAGCTGGCAAC-3′ (forward) and 5′-TCCAATCGACGGCTAGCTACC-3′ (reverse) [17] under the following conditions: (1) 94°C for 5 min, 1 cycle, (2) 94°C for 50 s, 65°C for 50 s, and 72°C for 1 min, 35 cycles, and (3) 72°C for 10 min, 1 cycle and stored at 4°C. The PCR product above was then diluted and used as the templates for the 471 bp fragment amplification with the primers 5′-CACCACGCCTGGCTAACTT-3′ (forward) and 5′-TCACGAGGTGGGCTGTTCT-3′ (reverse) under the following conditions: (1) 95°C for 3 min, 1 cycle, (2) 95°C for 30 s and 68°C for 1 min, 37 cycles, and (3) 72°C for 10 min, 1 cycle and stored at 4°C. The PCR products were subsequently treated according to the standard sequencing procedure of BigDye Terminator v3.1 Cycle Sequencing Kit in the PE Applied Biosystem (PE Applied Biosystem) using either the forward primer or the reverse primer. Electrophoresis was conducted on the ABI PRISM 3100 Genetic Analyzer (PE Applied Biosystem). ApoE-*ε*2/3/4 genotypes were determined by sequencing and restriction fragment length polymorphism (RFLP) as described in our earlier work [28].

### 2.4. Statistical Analysis

Genotype and allele frequencies of ApoE and promoter polymorphisms were determined in study groups. Hardy-Weinberg equilibrium and putative haplotypes estimation analysis for −491A/T, −427T/C, −219G/T, and ApoE-*ε*2/*ε*3/*ε*4 were calculated using the software program ARLEQUIN (version 2.0; Genetics and Biometry Laboratory, University of Geneva, Switzerland) [29]. All comparisons for differences of allele, genotype, and haplotype distributions among groups of MR, borderline MR, and controls were tested with Mantel-Haenszel *χ* ^2^ test using SAS 9.2 (SAS, Cary, NC). Finally, pairwise linkage disequilibrium coefficients (*D*′ and *r* ^2^) were calculated using the EMLD program developed by Qiqing Huang (http://www.mybiosoftware.com/population-genetics/4717).

## 3. Results

The distributions of gender, age, and counties did not differ significantly among MR, borderline MR, and controls (*P* > 0.05, Table 1). The individual allelic and genotypic frequencies of −491A/T, −427T/C, −219G/T, and ApoE-*ε*2/*ε*3/*ε*4 by study groups were shown in Table 2. The distributions of genotypes of all the selected loci were in Hardy-Weinberg equilibrium (*P* > 0.05). Comparisons among MR, borderline MR, and control groups with Mantel-Haenszel *χ* ^2^ test did not find any significant difference in allele or genotype distributions (*P* > 0.05). However, the comparison of the haplotype frequencies among three study groups revealed that (Table 3) haplotype between the promoter polymorphisms −491A, −427T, −219T, and *ε*4 was significantly associated with the phenotypes showing the high frequency in MR, lower frequency in borderline MR, and the lowest frequency in controls (Mantel-Haenszel *χ* ^2^ = 8.09, *P* uncorrected = 0.004). LD between all possible pairs of the four polymorphisms was calculated and the pairwise *D*′ and *r* ^2^ were shown in Table 4.

## 4. Discussion

In this study, we investigated if ApoE-*ε*2/3/4 and promoter polymorphisms of −491A/T, −427T/C, and −219G/T are associated with the risk of MR in children from the iodine deficiency area with high prevalence of MR. We did not find any significant association between individual variation at four common polymorphisms (−491A/T, −427T/C, −219T/G, and *ε*2/3/4) and MR or borderline MR. Interestingly, haplotype analysis showed that ATT*ε*4 is associated with increased risk of MR and borderline MR.

The physiological and pathological roles of ApoE in the central nervous systems are not entirely clear, but ApoE protein is produced in abundance in the brain by glia, macrophages, and neurons [30, 31]. It is well known that ApoE *ε*4 is a major genetic risk factor for late onset sporadic AD [10, 22] and has also been investigated for its association not only with dementia but also with normative cognitive development. Most studies have been conducted on adults, especially on older people, but much fewer studies have explored the relationship of ApoE and cognition in children. Several studies on ApoE genotype in relation to cognition in children showed inconsistent results. Most prior studies in school-aged children did not find any significant association with cognitive performance or school assessments, measured in different ways [5, 13, 14, 32], although one study detected a significant association with general cognitive factor [33] and ApoE *ε*4 predicted higher education in another study [34]. Cavani et al. investigated the association between ApoE genotype and MR in Down syndrome patients in 2000 and found no statistical differences between ApoE allele frequencies of Down syndrome, normal controls, and MR cases [35]. Our analyses based on individual locus found null associations with mental retardation, which is consistent with the prior studies with null associations. Interestingly, haplotype analysis suggested that ApoE may have relationship with intelligence in children. It may be very well helpful for future studies to include haplotype analysis.

To the best of our knowledge, our study is the first attempt to examine haplotype association of ApoE promoter and *ε*2/3/4 with intelligence in children. Therefore, our analyses should be considered as exploratory and hypothesis generating. The association should be interpreted with cautions although the significance can pass the Bonferroni correction (threshold = 0.005 = 0.05/10 tests). Due to moderate sample size, we could not exclude the possibility of chance finding. An alternative explanation for the positive association is that our subjects had some specialties compared to other studies. The samples in the study were recruited from the relatively isolated iodine deficiency area. The iodine deficient exposure may bring some new features to increase the feasibility of detection. The exon 3 of the ApoE gene possesses sequence homology with coding of the three major thyroid hormone plasma transport proteins (thyroid-binding globulin (TBG), transthyretin (TTR), and albumin) [36, 37]. If the ApoE genotypic variation affects the efficiency of transportation and metabolism of thyroid hormone and therefore influences neuronal cell growth during the first and second trimesters of fetal development [38, 39], the effect size of association between ApoE and intelligence can be modified by the exposure of iodine deficiency. This hypothesis needs confirmation from other studies. On the other hand, MR and borderline MR can be regarded as extreme outcomes of intelligence. Using the extreme phenotypes can increase the power to detect association.

The samples in the study were recruited from the relatively isolated Qinba mountainous area and we did not find a significant difference in allele frequencies among the two counties, which indicated lower risk of stratification bias. Due to the fact of poor education, less developed economy, and transportation, the area is almost isolated from other areas and has much less gene flow [28], so the subjects are helpful in controlling population stratification and have the advantage from the view of a genetic investigation [40, 41].

## 5. Conclusions

In summary, in this large population-based study, we did not find any significant association between single locus of the four common ApoE polymorphisms (−491A/T, −427T/C, −219T/G, and *ε*2/3/4) and MR or borderline MR in children. However, we found that the presence of ATT*ε*4 haplotype was associated with an increased risk of MR and borderline MR. Our present work may help enlarge our knowledge of the role of ApoE in cognitive functioning across the lifespan and the mechanisms of human cognition.

## Figures and Tables

**Table 1:** Population characteristics according to case and control.

	County	*N*	Sex ratio (F : M)	Mean age ± SD
Control	Zhashui	250	117/133	9.3 ± 2.8
	Ankang	84	37/47	

Border^a^	Zhashui	80	42/38	10.2 ± 2.9
	Ankang	44	22/22	

MR^b^	Zhashui	73	38/35	10.1 ± 2.8
	Ankang	57	27/30	

Sum		588	283/305	9.7 ± 2.9

^a^Borderline mental retardation (border) group.

^b^Mental retardation (MR) group.

**Table 2:** Distribution of allele and genotype across ApoE *ε*2/*ε*3/*ε*4,  −491A/T, −427T/C, and −219G/T polymorphisms in MR, borderline MR, and control.

Loci		Allele					Genotype			
	*N* ^a^	*ε*2	*ε*3	*ε*4	*P* ^b^	*N* ^a^	*ε*2+^c^	*ε*4+^c^	*ε*3/*ε*3	*P* ^b^

*ε*2/*ε*3/*ε*4										
MR	260	26 (23.4)	206 (21.4)	28 (26.9)	0.82	130	24 (23.4)	80 (20.3)	26 (28.3)	0.46
Borderline MR	248	20 (18.0)	211 (22.0)	17 (16.4)		123^a^	18 (18.0)	89 (22.5)	16 (17.4)	
Control	668	65 (58.6)	544 (56.6)	59 (56.7)		329^a^	53 (58.6)	226 (57.2)	50 (54.3)	

	*N* ^a^	T	A		*P* ^b^	*N* ^a^	T/T	T/A	A/A	*P* ^b^

−491A/T										
MR	260	3 (14.3)	257 (22.2)		0.21	130	0 (0)	3 (15.8)	127 (22.4)	0.45
Borderline MR	248	3 (14.3)	245 (21.1)			124	0 (0)	3 (15.8)	121 (21.3)	
Control	668	15 (71.4)	653 (56.7)			334	1 (100)	13 (68.4)	320 (56.3)	

	*N* ^a^	C	T		*P* ^b^	*N* ^a^	C/C	C/T	T/T	*P* ^b^

−427T/C										
MR	260	24 (21.8)	236 (22.1)		0.64	130	1 (14.3)	22 (22.9)	107 (22.1)	0.49
Borderline MR	248	20 (18.2)	228 (21.4)			124	0 (0)	20 (20.8)	104 (21.4)	
Control	668	66 (60.0)	602 (56.5)			334	6 (85.7)	54 (56.3)	274 (56.5)	

	*N* ^a^	G	T		*P* ^b^	*N* ^a^	G/G	G/T	T/T	*P* ^b^

−219G/T										
MR	260	81 (22.2)	179 (22.0)		0.77	130	16 (26.2)	49 (20.2)	65 (23.6)	0.78
Borderline MR	248	72 (19.8)	176 (21.7)			124	10 (16.4)	52 (21.5)	62 (18.9)	
Control	668	211 (58.0)	457 (56.3)			334	35 (57.4)	141 (58.3)	158 (57.4)	

^a^Counts of alleles or genotypes may not add up to total due to excluding the individuals genotyped as *ε*2/4.

^b^ *P* values are from Mantel-Haenszel *χ* ^2^ test in which MR, borderline MR, and control are ordinal variables.

^c^ *ε*2+: *ε*2/*ε*2 + *ε*2/*ε*3; *ε*4+: *ε*3/*ε*4 + *ε*4/*ε*4.

**Table 3:** Estimated haplotype frequencies for linkage disequilibrium among ApoE −491,  −427, −219, and *ε*2/*ε*3/*ε*4.

Haplotype^a^	Frequency	MR (*N* = 260)	Borderline MR (*N* = 248)	Control (*N* = 668)	Mantel-Haenszel *χ* ^2^	*P* value^b^	
						Uncorrected	Corrected
ATT*ε*2	0.06	0.07	0.02	0.06	0.0005	0.98	1.00
ATG*ε*3	0.19	0.19	0.22	0.18	0.37	0.54	1.00
ATT*ε*3	0.55	0.51	0.60	0.55	0.40	0.53	1.00
ATT*ε*4	0.07	0.10	0.07	0.05	8.09	0.004	0.02
ACG*ε*2	0.02	0.01	0.06	0.02	0.03	0.87	1.00
ACG*ε*3	0.06	0.08	0.01	0.07	0.07	0.80	1.00

^a^Haplotypes are from alleles of 4 polymorphisms: ApoE −491, −427, −219, and *ε*2/3/4. Only the haplotypes with frequency 0.01 and higher are shown.

^b^Corrected *P* value according to Bonferroni correction (6 tests).

**Table 4:** Pairwise linkage disequilibrium (*D*′/*r* ^2^) of −491A/T, −427T/C, −219T/G, and *ε*2/*ε*3/*ε*4 polymorphisms.

SNPs	LD estimate (*D*′ or *r* ^2^) for marker pair			
	−491A/T	−427T/C	−219T/G	*ε*2/*ε*3/*ε*4
−491A/T	—	0.921	0.241	0.216
−427T/C	0.002	—	0.895	0.589
−219T/G	0.001	0.171	—	0.286
*ε*2/*ε*3/*ε*4	0.001	0.075	0.049	—

The standardized *D*′ values are shown above the diagonal, and the *r* ^2^ values are shown below the diagonal.

## Abbreviations

ApoE: Apolipoprotein E
MR: Mental retardation
IQ: Intelligence quotient
NSMR: Nonspecific mental retardation
SNP: Single-nucleotide polymorphism
AD: Alzheimer's disease
RFLP: Restriction fragment length polymorphism.
